# Helical computerized tomography and NT-proBNP for screening of right ventricular overload on admission and at long term follow-up of acute pulmonary embolism

**DOI:** 10.1186/1757-7241-20-33

**Published:** 2012-05-04

**Authors:** Mia K Laiho, Veli-Pekka Harjola, Marit Graner, Anneli Piilonen, Merja Raade, Pirjo Mustonen

**Affiliations:** 1 Helsinki Malmi City Hospital, Department of Emergency care, POB 6501, 00099, Helsinki City, Finland; 2 Division of Emergency Care, Department of Medicine, Helsinki University Central Hospital, POB 340, 00029 HUS, Helsinki City, Finland; 3 Division of Cardiology, Department of Medicine, Helsinki University Central Hospital, POB 340, 00029 HUS, Helsinki City, Finland; 4 Department of Radiology, Helsinki University Central Hospital, POB 340, 00029 HUS, Helsinki City, Finland; 5 Red Cross Blood Transfusion Service, Kivihaantie 7, 00310, Helsinki, Finland

**Keywords:** Non-high risk pulmonary embolism, Follow-up, right ventricular dysfunction, NT-pro-BNP, Echocardiography, helical CT, CT pulmonary angiography

## Abstract

**Background:**

Right ventricular dysfunction (RVD) in acute pulmonary embolism (APE) can be assessed with helical computerized tomography (CT) and transthoracic echocardiography (TTE). Signs of RVD and elevated natriuretic peptides like NT-proBNP and cardiac troponin (TnT) are associated with increased risk of mortality. However, the prognostic role of both initial diagnostic strategy and the use of NT-proBNP and TnT for screening for long-term probability of RVD remains unknown. The aim of the study was to determine the role of helical CT and NT-proBNP in detection of RVD in the acute phase. In addition, the value of NT-proBNP for ruling out RVD at long-term follow-up was assessed.

**Methods:**

Sixty-three non-high risk APE patients were studied. RVD was assessed at admission in the emergency department by CT and TTE, and both NT-proBNP and TnT samples were taken. These, excepting CT, were repeated seven months later.

**Results:**

At admission RVD was detected by CT in 37 (59 %) patients. RVD in CT correlated strongly with RVD in TTE (p < 0.0001). NT-proBNP was elevated (≥ 350 ng/l) in 32 (86 %) patients with RVD but in only seven (27 %) patients without RVD (p < 0.0001). All the patients survived until the 7-month follow-up. TTE showed persistent RVD in 6 of 63 (10 %) patients who all had RVD in CT at admission. All of them had elevated NT-proBNP levels in the follow-up compared with 5 (9 %) of patients without RVD (p < 0.0001).

**Conclusions:**

TTE does not confer further benefit when helical CT is used for screening for RVD in non-high risk APE. All the patients who were found to have RVD in TTE at seven months follow-up had had RVD in the acute phase CT as well. Thus, patients without RVD in diagnostic CT do not seem to require further routine follow-up to screen for RVD later. On the other hand, persistent RVD and thus need for TTE control can be ruled out by assessment of NT-proBNP at follow-up. A follow-up protocol based on these findings is suggested.

## Background

Right ventricular dysfunction (RVD) is an independent and important predictor of early death in patients with acute pulmonary embolism (APE) [1-4].

Transthoracic echocardiography (TTE) is regarded as the most reliable method for assessment of RVD. On the other hand, low levels of brain natriuretic peptide (BNP) and N-terminal (NT)-proBNP seem reliable for ruling out RVD [5-10]. Combination of NT-proBNP with TTE may permit the identification of both non-high and high-risk patients with APE [10,11]. Currently, helical CT is the first-line imaging investigation in patients with suspected APE [12-15]. Helical CT can also be used to assess RVD. RVD detected by helical CT correlates with RVD determined by TTE [16]. RV enlargement on CT has been reported to predict early clinical outcome [17-20].

The data on the natural history of APE with respect to persistent RVD after hospital discharge is sparse. In study of Ribeiro et al in 1999 [21] an echocardiography performed six weeks after APE could identify patients with persistent RVD. According to our knowledge there are only a few prospective follow-up studies concerning non-high risk APE and in most of these studies RVD has been detected in the acute phase by TTE [22,23]. In a few follow-up studies diagnosis of initial RVD has been done by CT. Van der Meer et al. in 2006 [17] reported a three months follow-study in which RVD was assessed by CT at baseline. In that study RVD was found to predict mortality after initial hospitalization in patients with both massive and submassive APE. Golpe et al [24] followed APE patients for six months where the diagnosis was initially confirmed by CT. As far as we are aware there are no follow-up recommendations for patients with APE and RVD.

The aim of this study was to determine the role of helical CT and NT-proBNP in detection of RVD in the acute phase. In addition, the value of NT-proBNP for ruling out RVD at long-term follow-up was assessed.

## Methods

This study was conducted in Helsinki University Central Hospital, Helsinki, Finland between February 2003 and August 2004. Sixty-three consecutive patients of all diagnosed 261 patients at Emergency department (ED) were included in the study with a similar clinical management protocol. Subjects with high-risk APE (haemodynamically unstable patients), chronic pulmonary disease with regular ongoing medication, history of previous APE, ongoing anticoagulation therapy, end-stage cancer (estimated life expectancy less than seven months) were excluded from the study. There were several patients who were referred to the ED only for diagnostic investigations from the surrounding local hospitals and returned there for further treatment. There was also another PE study ongoing in hospital at the time of the present study. Those patients were excluded from the present study as well.

At admission RVD was assessed by CT and the patients were grouped as RVD positive and RVD negative. TTE was performed within 12 hours. ECG and samples for NT-proBNP and TnT were taken on admission. TTE, cardiac biomarkers and clinical evaluation were repeated seven months later.

A detailed medical history was recorded on admission including information on risk factors for thromboembolic events (such as age, gender, immobilization the preceding three months, hormone replacement or hormonal contraception therapy, family history of venous thromboembolism, active cancer, varicose veins, smoking and overweight).

A twelve-lead ECG was recorded on admission. Established criteria for RVD were used: T-wave inversion in leads V_1_ to V_3_, incomplete or complete right bundle branch block, S1Q3T3 pattern, and the presence of P pulmonale [25,26]. If any of these findings were present the patient was categorized as having RVD from the ECG.

An arterial blood gas analysis was performed on admission. Hypoxemia was defined as arterial pO_2_ below 11 kPa and hypocapnia as arterial pCO_2_ below 4.5 kPa. Blood pressure was measured and all patients underwent ultrasound examination of the lower limb veins.

Blood samples for plasma NT-proBNP, cardiac troponin T (cTnT) and D-dimer (fibrin degradation product) were collected on admission and analyzed using commercially kits: NT-proBNP (Roche Diagnostics Elecsys), cTnT (Roche Diagnostics Elecsys) and D-dimer (Roche Diagnostics Tina-Quant). An NT-proBNP value ≥ 350 ng/l was regarded as elevated. The cut-off level 350 ng/l was chosen because according to our local laboratory reference values ≥ 350 ng/ml are abnormal irrespective of age and sex. The assay specific cut-offs for cTNT (>0.03 ug/l) and for D-dimer (≥0.5 mg/l) were used. All the subjects were tested for thrombophilia in the routine tests of the local laboratory (activated protein C resistance, factor V Leiden mutation assay, prothrombin 20210 mutation assay, lupus anticoagulant testing, anticardiolipin IgG antibodies, beta2-glycoprotein 1 (beta2-GP1) antibodies, antithrombin activity, factor VIII activity, protein C activity and protein S activity).

The helical CT scan was performed as a part of the initial diagnostic work-up. Thirty-six CT examinations were performed with an 8-slice-scanner (GE Light Speed Ultra), 22 with a four-slice-scanner (22 with Philips Mx8000, one with GE High Speed) and five with a single slice scanner (GE High Speed LX/i). The volume of contrast material varied from 90 to 120 ml and was injected using bolus tracking with a power injector 4 ml/s. The collimation was 1 to 1.25 mm but 3 mm collimation with the single slice scanner. After the study period, all the CT scans were evaluated by two experienced radiologists who were blinded to the clinical findings. The left and right ventricular (LV, RV) diameters were measured on the axial image where the ventricles were their widest. The septum was evaluated as normal, straightened or bowed toward the left ventricle.

The criteria for RVD were RV/LV ratio over 1.0 and/or deviation (straightening or bowing) of the interventricular septum [16,27].

TTE was performed within 12 hours after the diagnosis of APE using the Vivid Digital Ultrasonography System (GE Vivid 5 or Vivid 7, Horten, Norway). Recordings on M-mode, two-dimensional color-flow, and Doppler were stored on a magneto-optic disc. Most of the patients were examined both in a left lateral and in a flat supine position. Parasternal and apical views were preferred. All the studies were performed by one of three experienced cardiologists. TTE was repeated seven months later by the same cardiologist as on admission. The analyses and measurements were carried out from stored data of the recordings and the investigators were blinded to all the clinical data.

Presence of RVD was assessed using the following established criteria: 1) the ratio of RV and left ventricular end-diastolic diameter (RV/LV) above 1.0, 2) the presence of paradoxical septal wall motion, and 3) mean TR peak velocity greater than 2.8 m/s. At least one of these criteria had to be positive.

Statistical analyses were performed with SPSS 15.0 for Windows (SPSS Inc., Chicago, IL, USA). The subjects were categorized according to CT findings into two groups: RVD positive and RVD negative. Data are presented as frequencies or percentages for categorical variables and as mean (SD) for continuous variables, unless otherwise noted. Normality of continuous variables was checked by the Kolmogorov-Smirnov test. Between-group differences were assessed by the Mann–Whitney U-test. Categorical data were compared by the chi-square test or Fischer´s exact test. P-values <0.05 were considered statistically significant.

This study was approved by the Ethics Committee of the Helsinki University Central Hospital, and written informed consent was obtained from all participants.

## Results

The clinical characteristics of the patients (30 males and 33 females) with a mean age of 55 years, range 18 to 86 years, and risk factors for venous thromboembolism are represented in Table 1. Risk factors for venous thromboembolism were similar in RVD negative and positive groups (data not shown). No patient had an acute coronary syndrome according to established clinical criteria. All the patients had normal left ventricular systolic function in TTE.

**Table 1:** Characteristics and risk factors of 63 patients

**Characteristics and risk factors of 63 patients**	
**Variable**	**Patients, n (%)**
Age >75 years	10 (16)
Gender	
Male	30(48)
Female	33 (52)
Body mass index >25 kg/m^2^	50 (79)
Immobilization	28 (44)
(prolonged immobility, hospitalization, surgery within 3 months)	
HRT *	7 (11)
Hormonal contraception	6(10)
History of DVT**	7 (11)
Family history of VTE***	5 (8)
Thrombophilia****	9 (14)
Active cancer	3 (5)
Varicose veins	27 (43)
Diabetes mellitus	5 (8)
Smoking	
Smoker	11 (17)
Ex-smoker	19 (30)
Non-smoker	33 (52)

* HRT = Hormone replacement therapy.

**DVT = Deep venous thrombosis.

*** VTE = Thromboembolic disease.

**** Known previously or detected in clinical laboratory tests.

All the patients were initially treated in hospital and stayed in an observation unit if clinically necessary. Three patients in the RVD positive group received thrombolysis as a 2-hour alteplase infusion. All the patients received subcutaneous low molecular weight heparin in a weight-adjusted dose followed by warfarin for at least six months. There were no bleeding complications. All the patients survived until the seven months follow-up visit. The mean initial systolic blood pressure was 140 (SD 21) and diastolic 83 (SD 13) mmHg.

**Figure 1:**
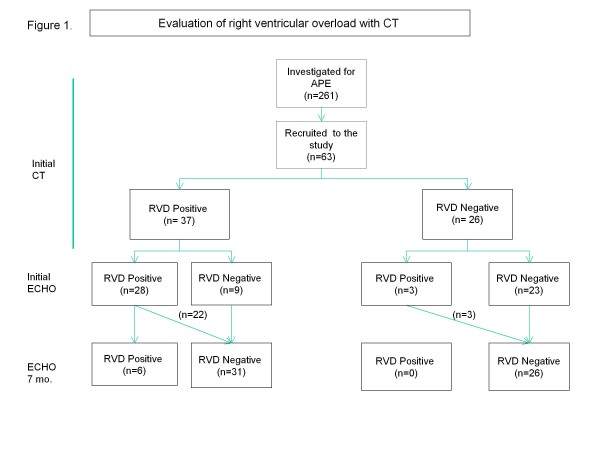
**Evaluation of right ventricular overload with CT.** Computerized tomography (CT) Right ventricular dysfunction (RVD) Echocardiography (ECHO).

Right ventricular overload was detected with CT in 37 patients (59 %). (Figure 1).

Both increased RV/LV ratio and septum deviation were observed in 27 (73 %) patients whereas only septum deviation was observed in 7 (19 %) patients and increased RV/LD ratio in 3 (8 %).

Twenty-eight patients (76 %) in the RVD positive group also had signs of RVD in TTE. Nine of the RVD positive patients (24 %) showed no echocardiographic alterations of RV morphology or function. Overall findings of RVD in CT correlated strongly with those in echocardiography (p < 0.0001). Findings suggesting RVD were found with TTE in three of the initially RVD negative patients. The only sign of RVD in these patients was as a slightly increased mean tricuspid regurgitation peak velocity of 2.9-3.1 m/s.

Of the patients who had RVD in CT 32 patients (86 %) had elevated NT-proBNP level in contrast to only 7 patients (27 %) of patients without RVD (p < 0.0001). TnT was above the reference value in 11 patients (17 %), nine of them (82 %) in RVD positive and two (18 %) in RVD negative group( p < 0.082 ).

Signs of RV overload in the ECG on admission were detected in 32 (51 %) patients. In the RVD positive group 24 patients (87 %) had signs of RV overload in the ECG whereas eight patients (13 %) had such findings in the RVD negative group (p < 0.0001).

Arterial blood gas analysis was available in 60 patients. Arterial hypoxemia was detected in 56 (93 %) and hypocapnia in 27 (45 %). RVD positive patients (n = 37) had hypoxia and hypocapnia (n = 36) significantly more often than the RVD negative group patient (p < 0.0001). The elevated D-dimer level was detected in all patients. The mean levels of D-dimer did not differ between the study groups; 10 (SD 7) mg/l in the RVD and 7 (SD 7) mg/l in the RVD negative group (p < 0.102). The prevalence of DVT was similar in both groups ; DVT was diagnosed in 32 patients (51 %), 18 (56 %) in the RVD positive and 14 (44 %) in the RVD negative group (p > 0.05).

At the seven-month follow-up persistent RVD was detected by echocardiography in 6 (10 %) of all the patients. CT detected every patient who had persistent RVD at 7 months, a sensitivity of 100 %, 95 % CI (54 %, 100 %). All these 6 patients had elevated NT-proBNP levels in the follow-up compared with 5(9%) patients without RVD (p < 0.0001). All the patients who were found to have persistent RVD, had RVD in both CT and TTE at admission. On the other hand the lack of RVD in CT at admission predicted normal RV function at follow-up in 26 out of 57 patients, with specificity of 46 %,(95 % CI 32-, 59 %).

## Discussion

This prospective follow-up study of non-high risk APE patients shows that helical CT is a practical and valuable method for detection of RVD in clinical practice. Furthermore, normal RV findings in CT as well as NT-proBNP level ≤ 350 ng/l excluded RVD both in the acute phase and at seven months follow-up.

In our study RVD was detected with CT in 59 % of patients with non-high risk APE. In earlier CT imaging studies which have included both massive and non-massive PE, RVD has been reported in 58 % to 64 % of patients [16,17,28]. Our results also are in agreement with earlier studies in which RV dysfunction has been detected with TTE in approximately 50 % of patients [1,3,28-30]

There were 9 patients in whom RVD was observed in CT but not in TTE. This could be owing to the difference in timing of the investigations. CT was performed as a routine diagnostic imaging study of APE in the ED whereas TTE was recorded within 12 hours. It is possible that some minor RVD changes had already normalized within 12 hours after initializing the treatment for APE. The patients who had signs of RVD in TTE but not in CT had only borderline increase in the maximal velocity of TR, a finding which may be considered unspecific.

Our study demonstrates that NT-proBNP is valuable in ruling out the probability of RVD. Previous studies have indicated that NT-proBNP may be useful in the identification of non-high and high-risk patients and low plasma NT-proBNP levels predicts an uncomplicated clinical hospital course in APE patients [6,8]. In the present study, however, the hospital course of all the patients was uncomplicated regardless of the NT-proBNP level, and all study patients survived throughout the follow-up time. Thus the role of NT-proBNP for predicting mortality risk could not be evaluated in the present study. However, level of NT-proBNP under ≤ 350 ng/l at admission excluded development of persistent RVD during follow-up. A normal NT-proBNP at follow-up also was a valuable tool for screening out RVD during follow-up; none of the patients with a normal NT-proBNP were found to have persistent RVD.

The International Collaborative Study on NT-proBNP has established cut-off values for NT-proBNP in acute heart failure [31-34]. According to this study an NT-proBNP value below 300 ng/ml could be used to rule out heart failure. On the other hand studies in APE have used 500 ng/l as a cut-off for RVD [35] and 1000 ng/l[10] which also predict unfavorable outcome in PE. In our study the upper limit of the local normal range, i.e. 350 ng/l, was chosen as the cut-off level. One can assume that a lower cut-off might increase sensitivity but decrease specificity of the marker.

TnT -values did not differ significantly between the RVD positive and negative groups which was expected result since high-risk PE patients were excluded from the study. Acute coronary syndrome was excluded from all the patients using established clinical criteria.

The ECG and arterial blood gas analysis proved to be valuable tools when assessing the probability of RVD in acute PE. Patients with RVD showed significantly more often both ECG findings and abnormal gas analysis compared to those without it.

One may consider performing CT and TTE not exactly simultaneously as a limitation to the study. However, this most often is the case in clinical practice and also in previous studies (24). Moreover, the primary purpose of this study was not to compare CT and TTE with each other.

At seven-month follow-up only TTE was done in order to avoid radiation. None of the patients died during the follow-up. On the other hand we did not aim to analyze prognostic effects on mortality since the limited number of patients does not allow further conclusions on survival. The present study found persistent RVD in 10 % of all study patients at seven-month follow-up. Earlier follow-up studies have detected RVD in 6 to 20 % of patients [24,36]. In our study every patient with RVD at follow-up also had RVD in the initial CT and TTE. On the other hand none of the patients with a normal NT-proBNP at follow-up had persistent RVD.

In study by Pengo et al [37] the investigators found symptomatic chronic thromboembolic pulmonary hypertension (CTPH) in 4 percent of patients after two years of first episode of symptomatic pulmonary embolism. Thus, the incidence of CTPH is not rare. According to our knowledge there are not available systematic follow-up protocols for APE patients. However, in clinical practice there is a real need for them.

## Conclusions

In conclusion, the present study shows that CT is a practical and valuable imaging method for detection of RVD in non-high risk APE patients. In this study all the patients who were found to have RVD at the seven months follow-up also had RVD in the acute phase CT and TTE. Furthermore, NT-proBNP level ≤ 350 ng/l excluded RVD at the seven months follow-up.

According to these results we suggest a follow-up protocol, in which patients with RVD on admission CT are rescreened during the follow-up by measuring NT-proBNP levels. If the levels are elevated an echocardiography is performed. Timing of the follow-up visit and NT-proBNP rescreening to the point of the planned discontinuation of anticoagulation treatment might be appropriate. Alternatively, if anticoagulation therapy is to be permanent then biomarker screening might be done from six to twelve months after APE. This suggested follow-up protocol needs to be validated in a larger patient population.

## Competing interests

V-PH is a consultant for Roche Diagnostics. AP has been in international congress funded by Eli Lilly and has given educational lecture for Eli Lilly and Astra Zeneca. The other authors declare no competing interests.

## Authors’ contributions

MKL: Design of the study, collected and analyzed the data and drafted the manuscript. V-PH: Design and coordination of the study, collected the data and revised the manuscript. MG: Design of the study, collected and analyzed data and revised the manuscript. AP: design of the study, analyzed and revised manuscript. MR: analyzed data and revised manuscript. PM: design and coordination of the study, collected the data and revised the manuscript. All authors have read and accepted the manuscript.
